# Cavity Effect of Gold Nanoparticles on Mid-Infrared Light

**DOI:** 10.1021/acsomega.4c10454

**Published:** 2025-03-21

**Authors:** Wenjie Yu, Cunliang Yang, He Min, Haipeng Liu, Yufeng Ma, Zhiheng Yu, Shuo Yuan, Heshuang Dong, Ke Wang, Bo Song, Jijun Feng

**Affiliations:** † Shanghai Key Laboratory of Modern Optical System, Engineering Research Center of Optical Instrument and System (Ministry of Education), School of Optical-Electrical and Computer Engineering, University of Shanghai for Science and Technology, Shanghai 200093, China; ‡ The Key Laboratory of Medical Electronics and Digital Health of Zhejiang Province, Jiaxing Nanhu University, Jiaxing, Zhejiang 314001, China

## Abstract

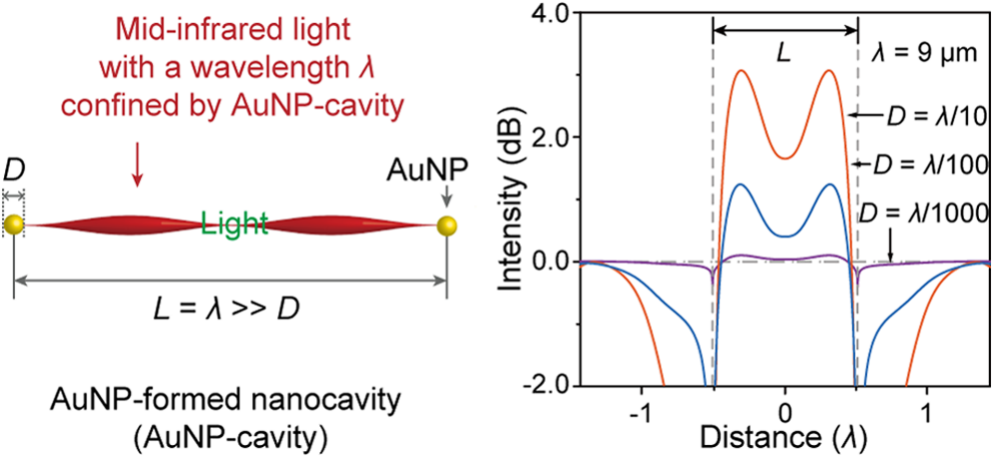

Nanophotonics has attracted wide attention in photonic devices and biotechnology. Interaction of visible and near-infrared lights with metal nanoparticles (NPs) is already well explored, leading to a mount of applications, especially in high-sensitivity biodetection. However, the effects of metal NPs on mid-infrared (MIR) light are still lacking because the light cannot resonantly excite the surface electron oscillation of the NPs. Recently, gold NP (AuNP)-assisted experiments indicate that AuNPs can be used in the detection of MIR biophotons, but the underlying mechanism remains unclear. Here, constructing a cavity by two AuNPs and performing finite difference time domain simulations based on Maxwell equations, we demonstrate that even if the AuNP dimension is significantly smaller than the MIR wavelength, the AuNP-formed cavity (AuNP-cavity) still can confine the light. The confinement effect increases with an increase in the wavelength or the cavity length when the cavity length and wavelength are fixed, respectively, while it vanishes only when the AuNP dimension is less than 1000th of the light wavelength. These results can be attributed to the resonance of MIR light with the two AuNPs, and in this view, it can be said that this nanocavity overcomes the diffraction limitation of the optical system. Our findings provide an understanding of the biophoton detection mentioned above, potentially promoting the applications of metal NPs in biotechnology and even in MIR-related imaging and wave-guiding circuits.

## 1 Introduction

Nanophotonics has attracted wide attention in the fields of photonic devices and biotechnology. By optimizing nanomaterials and designing nanostructures, various optical modes are deeply explored, which enables the precise control of light propagation at the nanoscale by finely tuning the interaction between light and matter.^1,2^ The advancements have not only broken the diffraction limit^3,4^ but also greatly improved the overall performance of optical systems, which leads to the wide applications including biodetection,^5−8^ environmental monitoring,^9,10^ and laser manufacturing.^11,12^

Due to the excellent optical properties and biocompatibility of gold nanoparticles (AuNPs), this type of nanostructure has been widely employed in high-sensitivity biodetection. When incident light resonates with the surface electron oscillation of AuNPs and induces surface plasmon resonance (SPR), the absorption and scattering of light are dramatically enhanced, particularly in the wavelength region of 400–800 nm with the peaks located at ∼520 nm.^13,14^ Thereby, AuNP SPR can significantly amplify the light field as well as the interaction of light and matter, which is employed in the sensitive detection of molecules.^15^ Moreover, in the experiments of surface-enhanced Raman scattering (SERS) with visible and near-infrared lights, AuNPs are proven to have excellent abilities at detecting low concentrations of molecules.^16−19^ In addition to the light in the above and ultraviolet ranges, recent studies suggest that mid-infrared (MIR) light also plays crucial roles in biological processes, expanding the definition of biophotons. For instance, a 53.7 THz MIR light can enhance the activity of K^+^ ion channels with the help of resonant effects, affecting neuronal action potentials and modulating animal behaviors.^20−23^ This new progress is expected to be used in biodetection, especially biophoton detection. A challenge in the researches is how to detect ultraweak MIR light because the photonic energy is significantly lower than that of visible and NIR lights, and there is a lot of surrounding environment noise in the detection.^24^ AuNP-assisted polymerase chain reaction (PCR) very recently showed that the PCR efficiency periodically oscillated as the AuNP concentration increased.^25^ After the Fourier transform, the wavelength of photons released in the PCR process was determined to be ∼9 μm. Such a detection cannot be understood by conventional SPR because MIR light, as a lower-frequency light, cannot effectively excite free electrons of AuNPs, while in the MIR range, the dielectric constant greatly increases, enhancing the loss and thus limiting the resonance effect.

**Figure 1:**
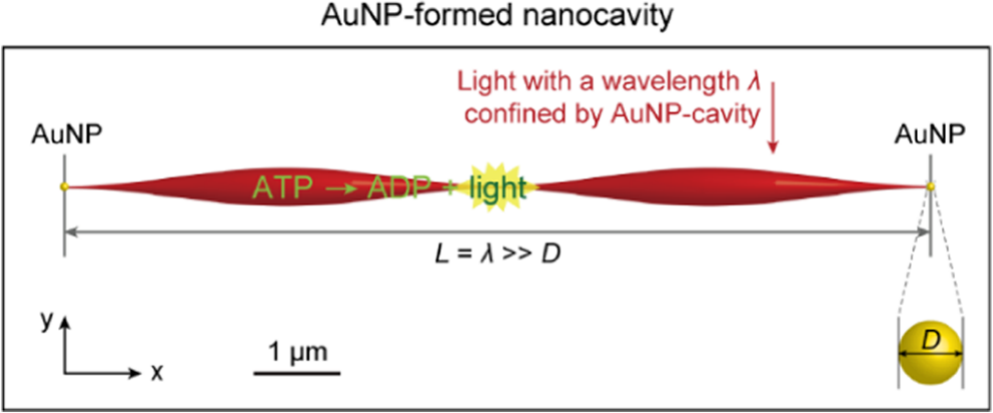
Schematic representation of a AuNP-formed cavity (AuNP-cavity) overcoming the diffraction limitation. The AuNP diameter (*D*) is significantly less than the wavelength (λ = 9 μm) of MIR light. When the cavity length (*L*) equals an integer multiple of the half-wavelength λ/2, the AuNP-cavity can confine the MIR light, i.e., it overcomes the diffraction limitation (*D* > λ) of an optical system.

In this study, using two AuNPs to construct an optical nanocavity (AuNP-cavity), by the finite difference time domain (FDTD) simulations based on Maxwell’s equations, we investigated the confinement effect of AuNPs on MIR light with a micron-scalar wavelength (Figure 1). Although the diffraction limitation is obviously beyond, i.e., the diameter (*D*) of AuNPs clearly less than the light wavelength (λ), the AuNP-cavity still can confine the light in the case of *D* > λ/1000. The confinement effect increases with an increase in the light wavelength or in the cavity length when the cavity length and wavelength are fixed, respectively. These results can be attributed to the resonance of MIR light with the two AuNPs. The findings provide a potential explanation for the previous AuNP-assisted PCR detection of the biophotons.

## 2 Results and Discussion

**Figure 2:**
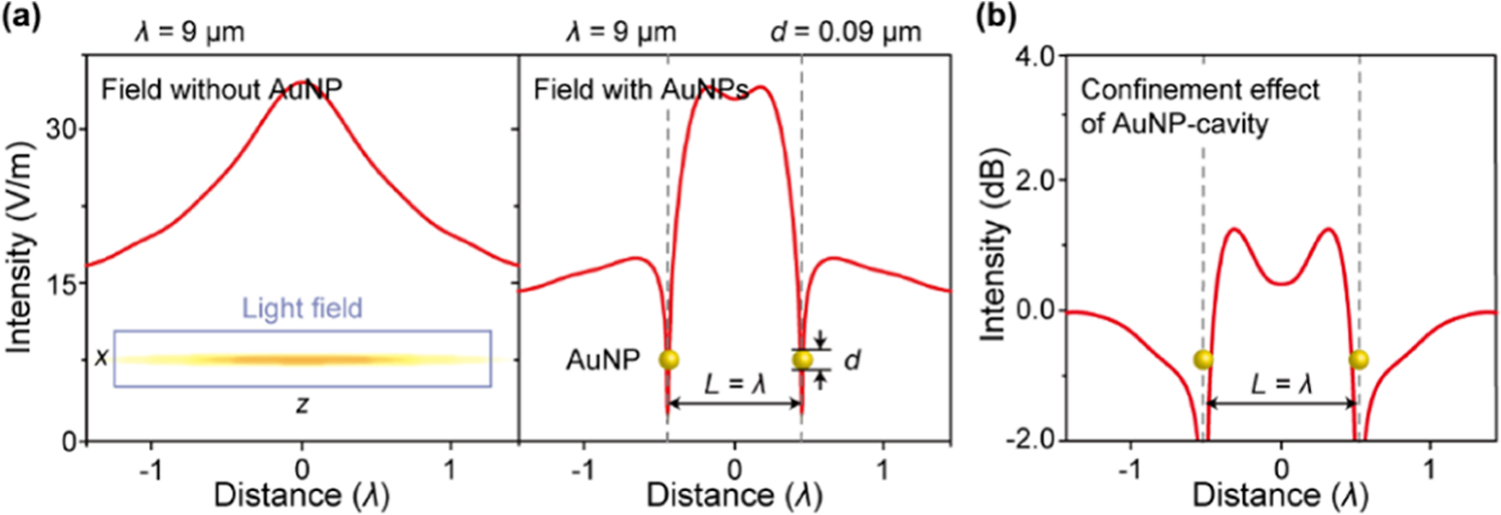
Mid-infrared field distributions with the absence and presence of the AuNP pair. (a) AuNP-absence (left, *I*_0_) and AuNP-presence (right, *I*) field intensities. Inset of the left panel: typical two-dimensional distribution of the field. The red-to-yellow ribbon (red: high intensity; yellow: low intensity) and golden balls indicate the light field and AuNPs, respectively. The labels λ, *d*, and *L* denote the light wavelength, AuNP diameter, and inter-AuNP distance, respectively. (b) Relative field intensity by an equation *I*_rel_ = 10 log_10_ (*I*/*I*_0_).

Two AuNPs were introduced to build an optical nanocavity of MIR light, and their distance was labeled as *L*. (Figure 1). For simplicity, the surrounding environment was set to a vacuum with the medium’s refractive index *n*_g_ = 1. Two electric dipoles were symmetrically positioned at a height of ±1 μm to form a source for simulating the MIR light released by the hydrolysis of adenosine triphosphate (ATP) between AuNPs in the experiments^26^ (Figure S1). The simulated light is thus a diverging wave (not strictly parallel) with a peak energy at the center (Figure 2a, left). The effect of AuNP-cavity on the light field was investigated based on Maxwell’s equations, which is the cornerstone of electromagnetic theory describing the electric (**E**) and magnetic (**B**) fields as follows
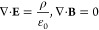

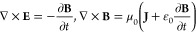
where ρ, **J**, *ε*_0_, and μ_0_ indicate the charge density, current density, permittivity, and permeability, respectively. Further, with the help of the FDTD simulations, we obtained the spatial distribution in the intensity of the electromagnetic field (i.e., light). To highlight the effect of AuNPs, the relative intensity (*I*_rel_) (Figure 2a, left) was applied with a unit of decibel (dB), i.e., subtracting the original field intensity (*I*_0_) without AuNPs (Figure 2a, left) from the field intensity (*I*) with AuNPs (Figure 2a, right) by an equation *I*_rel_ = 10 log_10_ (*I*/*I*_0_). More details of the methods and simulations are presented in the Supporting Information.

### 2.1 Effect of AuNP Size on the Confinement of MIR Light

**Figure 3:**
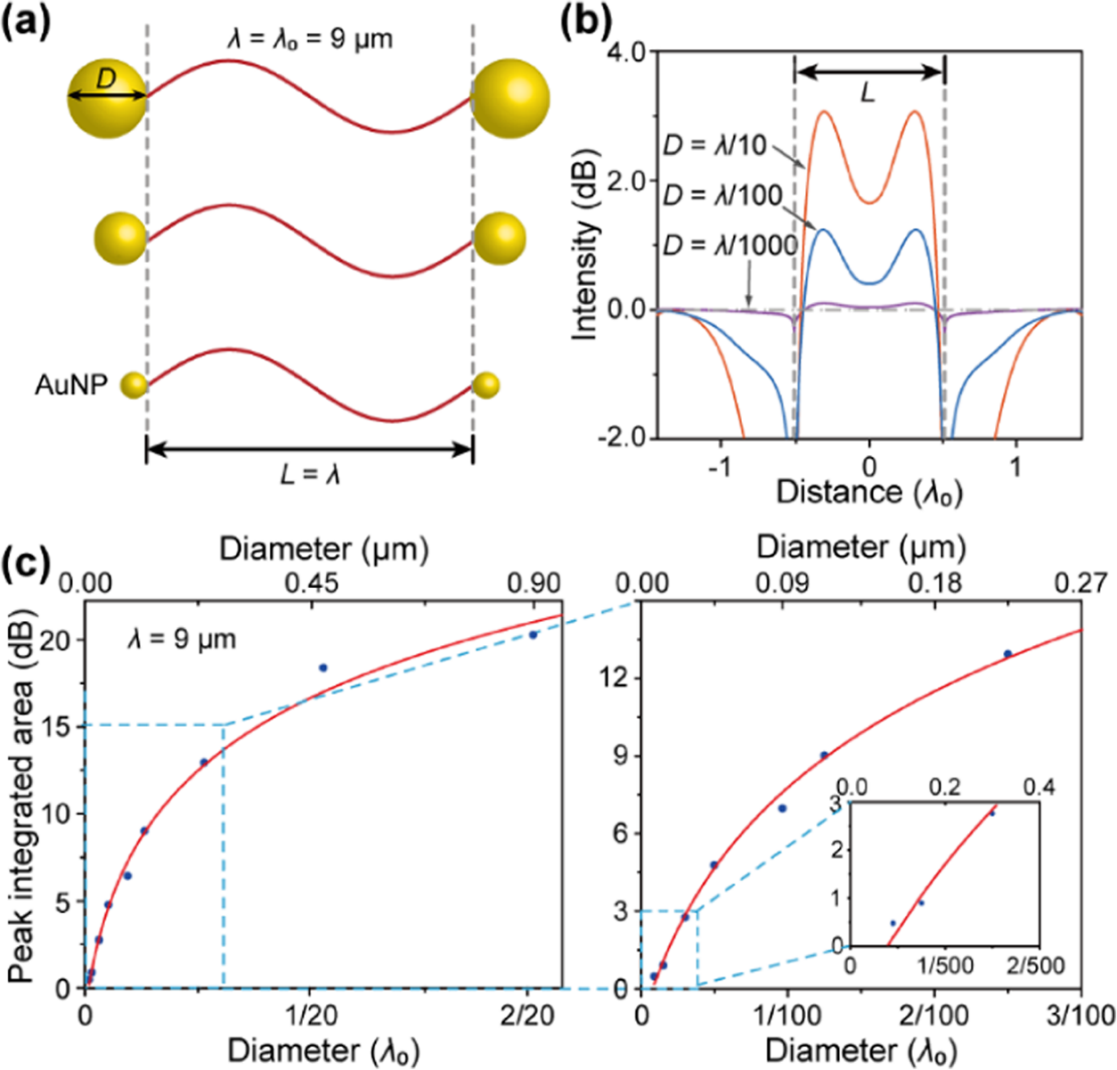
Dimension effect of gold nanoparticles on the ability of AuNP-cavity confining mid-infrared light. (a) Schematic of AuNP-cavity with a gradually decreasing AuNP diameter (*D*). The labels λ and *L* indicate the light wavelength and inter-AuNP distance, respectively, while λ_0_ = 9 μm denotes a fixed wavelength, as taken as a dimension unit. (b) Typical intensity distribution of the light field confined by the nanocavity. (c) Peak-integrated area (PIA) of the AuNP-cavity confined light with respect to the AuNP diameter.

To explore the effect of AuNP size on the confinement of MIR light by a AuNP-cavity, we performed simulations of a MIR electromagnetic field (EMF) between two AuNPs with different AuNP diameters. We used a MIR wavelength of 9 μm, and set the cavity length *L* to the wavelength first (Figure 3a). The cases with AuNP diameters of *D* = λ/10 (i.e., 0.9 μm) to λ/1000 (i.e., 0.009 μm) were studied. The typical confinement peaks of light are shown in Figure 3b. When *D* = λ/10, λ/100, and λ/1000, two peaks with identical locations appeared in the nanocavity, and the strength gradually weakened but remained above zero. Therefore, although the AuNP dimension is significantly less than the MIR wavelength, the AuNP-cavity still can confine the MIR light. To quantitatively study the confinement effect of AuNP-cavity, we introduced a peak-integrated area (PIA) by an integral over the relative field intensity of the peak. The calculated results are shown in Figure 3c. As the AuNP diameter decreased from λ/10 to λ/1000, PIA logarithmically decreased from 19.75 to 0.48, with the slow and sharp decreases when *D* > λ/100 and *D* < λ/100, respectively. Therefore, the confinement effect of AuNP-cavity on MIR light decreases as the AuNP diameter decreases, and the substantial dimension effect of AuNP majorly occurs in the region of *D* < λ/100 (Figure 3c, right). RPA was ignorable as *D* < λ/1000, indicating that the confinement effect of AuNP-cavity is vanishing for *D* < λ/1000, i.e., a critical value of the AuNP diameter occurs at *D* ≈ λ/1000 (Figure 3c, right inset). We thus can conclude that the AuNP-cavity still has the confinement effect on MIR light even if the AuNP dimension is clearly smaller than the light wavelength, and this effect vanishes only when the AuNP diameter is much smaller than the wavelength, i.e., *D* < λ/1000.

### 2.2 Effect of Wavelength on the MIR Light Confinement by AuNP-Cavity

**Figure 4:**
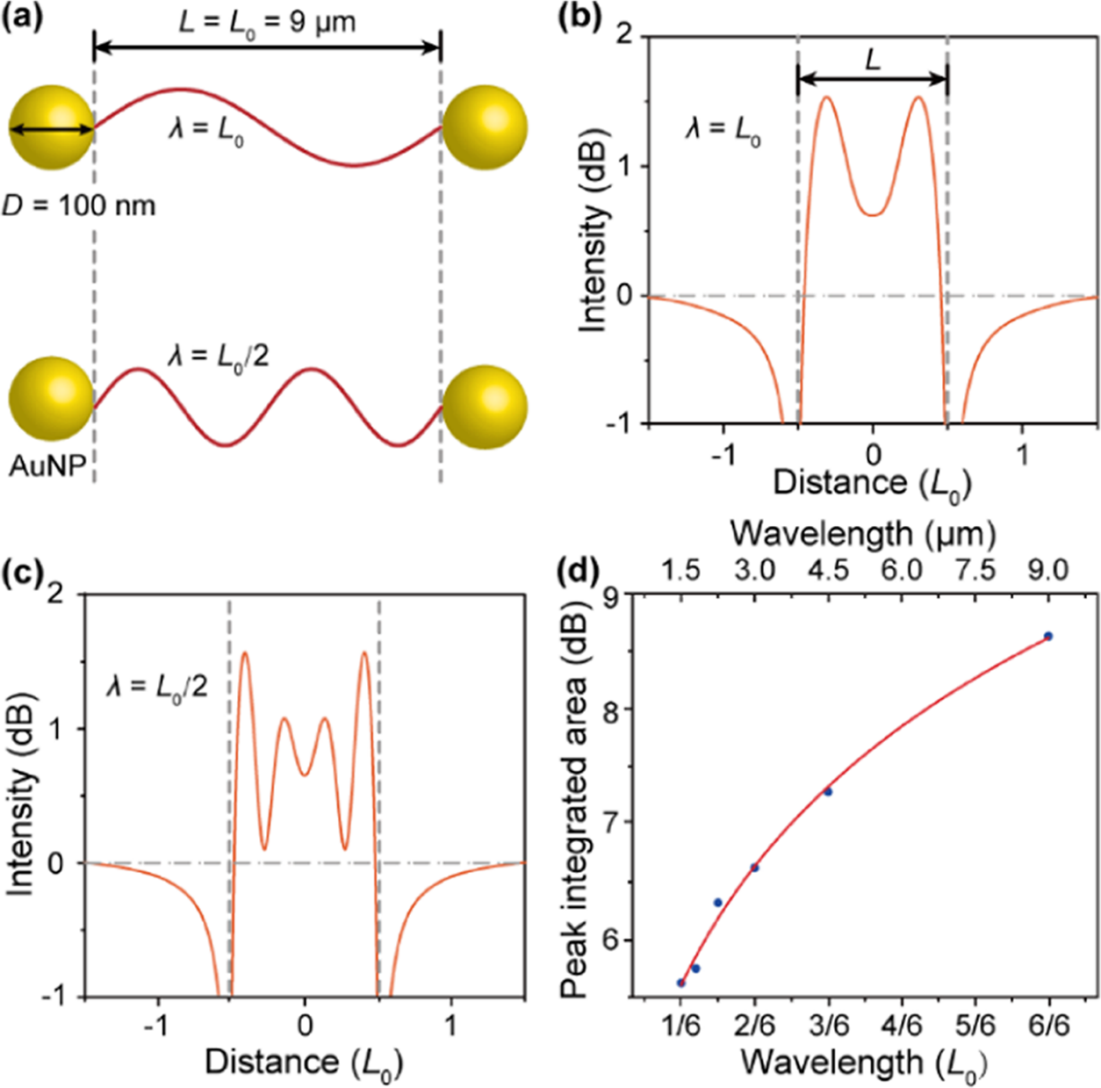
Wavelength effect of mid-infrared light on the confinement of the light by AuNP-cavity. (a) Schematic diagram of AuNP-cavity with a gradually decreasing light wavelength (λ). The label *L*_0_ = 9 μm denotes the fixed cavity length (*L*), which is taken as a dimension unit. (b, c) Typical intensity distribution of the light field resonantly confined by the cavity. (d) Peak-integrated area of AuNP-cavity confined light with respect to the light wavelength.

To explore the effect of wavelength on the MIR light confinement by the AuNP-cavity, we conducted simulations of MIR EMF between two AuNPs with a fixed AuNP diameter and different MIR wavelengths. A cavity length of *L* = 9 μm and a AuNP diameter of *D* = 100 nm were applied, while the wavelength λ was varied from *L* (i.e., 9 μm) to *L*/6 (i.e., 1.5 μm) (Figure 4a). The typical confinement peaks are shown in Figure 4b,c. At λ = *L*, two peaks with an intensity of 1.55 were observed, indicating that the MIR EMF is confined in the cavity. At λ = *L*/2 (i.e., 4.5 μm), four evenly spaced resonance peaks appeared, denoting that the MIR EMF generates four confinement peaks. To study the confinement effect of the AuNP-cavity on MIR light quantitatively, we calculated the peak-integrated area. As the wavelength increased from *L*/6 to *L*, the PIA logarithmically increased from 5.63 to 8.63 dB (Figure 4d). We thus can conclude that an increase in the wavelength still enhances the confinement effect of the AuNP-cavity on the MIR light, though it reduces the number of confinement peaks.

### 2.3 Effect of Cavity Length on the MIR Light Confinement by a AuNP-Cavity

**Figure 5:**
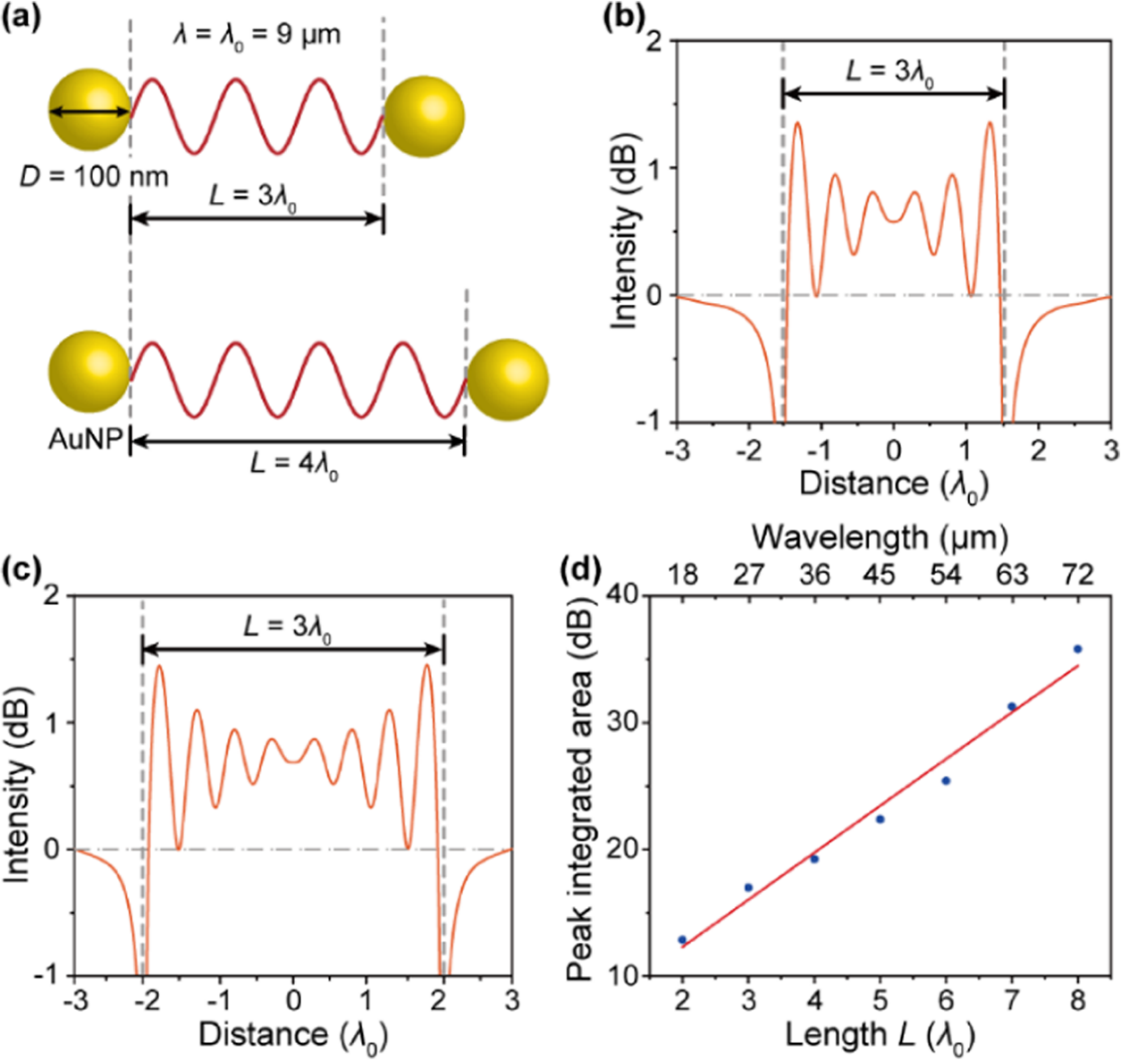
Effect of cavity length on the confinement of mid-infrared light by AuNP-cavity. (a) Schematic diagram of the AuNP-cavity with a gradually decreasing nanocavity length (*L*). The label λ_0_ = 9 μm indicates the fixed wavelength that is taken as the dimension unit. (b, c) Typical intensity distribution of the light field confined by the cavity. (d) Peak-integrated area of the AuNP-cavity confined light with respect to the cavity length.

To explore the effect of cavity length on the MIR light confinement by a AuNP-cavity, we carried out simulations of MIR EMF between two AuNPs at a fixed AuNP diameter with different cavity lengths. We applied a wavelength of λ = 9 μm and a AuNP diameter of *D* = 100 nm, while the cavity length *L* was varied from 2λ (i.e., 18 μm) to 8λ (i.e., 72 μm) (Figure 5a). The typical confinement peaks are shown in Figure 5b,c. At *L* = 3λ, six peaks with equal spacing and a maximum intensity of 1.37 dB were observed, indicating that the MIR EMF is confined in the cavity. A similar effect, with eight peaks, appeared at *L* = 4λ. The relationship of PIA with the cavity length is shown in Figure 5d. As the cavity length increased from 2λ to 8λ, PIA logarithmically increased from 12.86 to 35.81 dB. Therefore, we can conclude that increasing the AuNP-cavity length increases both the number of confinement peaks and the intensity of confined light, thus enhancing the cavity’s confinement effect.

### 2.4 Mechanism Underlying the Confinement AuNP-Cavity on MIR Light

The resonance effect is key to understanding the mechanism underlying the MIR light confinement of the AuNP-cavity. For Maxwell’s equations, there are plane wave solutions of electric and magnetic fields, **E**(**r**, *t*) = **E**_**0**_ exp[*i*(**k**·**r** – *ωt*)] and **B**(**r**, t) = **B**_**0**_ exp[*i*(**k**·**r** – *ωt*)], with |**k**| = 2π/λ and ω = 2π*ν*, where λ and *ν* mean the wavelength and frequency, respectively. When the inter-AuNP distance *L* satisfies the resonance condition *L =* λ*q*/*2n* (*n*: the refractive index, *q*: a positive integer), two counterpropagating waves superpose to form a standing wave mode between two AuNPs of AuNP-cavity, i.e., the light is confined, leading to the confinement peaks in the previous simulations. Such a resonant mode will appear even if the AuNP diameter is much smaller than the light wavelength. The diffraction limit refers to the physical limit of an optical system’s resolution, and in simple terms, it means whether light can “see” an object. For a conventional optics system, the mirror usually has a diameter of the centimeter level, no matter how thin the metal coating of mirror is, the light always can “see” the mirror (Figure S2). In contrast, for a AuNP with a diameter much smaller than a wavelength, the huge size difference will prevent the light from “seeing” the AuNP, and the light will bypass it, continuing to propagate along the original trajectory; i.e., the AuNP effect can be ignored. However, in a system of two AuNPs (e.g., AuNP-cavity), as indicated by our studies above, it is feasible to form a resonance of light with the AuNPs, which can confine the light; i.e., the effect of two AuNPs cannot be ignored. We thus can conclude that in the view of resonance effect, the diffraction limit is beyond.

### 2.5 Relationship with the AuNP-Enhanced PCR Experiments

In the AuNP-PCR biodetection experiment,^27^ the AuNP concentration is adjusted to achieve different distances between two AuNPs (see the detailed discussion in the Supporting Information), and the PCR efficiency is recorded as distance-dependent oscillation signals (Figure S3). Fourier transform converts the signals from real space to wavelength domains, resulting in a spectrum where peaks correspond to specific wavelengths of light. The major peak is located at ∼9 μm, indicating that there is an MIR light with a wavelength of 9 μm. According to our studies above, a standing wave of light forms when the inter-AuNP distance is an integer multiple of the half-wavelength λ/2. Namely, the photons (i.e., ultraweak light) are confined in the space between the AuNPs, limiting the radiation dissipation of ATP-released energy in the AuNP-PCR experiments and thus enhancing the energy-utilization efficiency of DNA extending. In contrast, when the inter-AuNP distance is an odd multiple of λ/4, the standing wave and the cavity-confinement effect of light energy vanish, leading to the DNA-extending efficiency going back to the level without AuNPs, as corresponds to our case of the original field. It is worth noting that when only one AuNP is considered, the huge difference in AuNP size and MIR wavelength will prevent the MIR light from “seeing” the AuNP. Almost all of the light thus will bypass the AuNP and continue to propagate along the original trajectory, leading to the fact that the effect of one AuNP on the light is usually ignored if no quantum excitation, e.g., the explanation of single-stranded DNA binding for the AuNP-enhanced experiments.^23,−34^ When two AuNPs are considered with an interparticle distance matching the previous resonance condition, the confinement effect of two AuNPs on the light cannot be ignored, though it is very weak, because it can be amplified of ∼2^*N*^ by cascade reactions, e.g., the PCR.

## 3 Conclusions

In summary, by the simulations and analyses based on Maxwell equations, we found that a AuNP-cavity can overcome the diffraction limitation of the system and exhibit a significant confinement effect on MIR light when the AuNP dimension is greater than 1000th of the wavelength. The confinement effect increases with an increase in the wavelength or in the cavity length when the cavity length and wavelength are fixed, respectively. The observations can be attributed to the resonance of MIR light with the two AuNPs. These results provide an optical understanding of the periodic oscillation of PCR efficiency observed in the AuNP-assisted PCR experiments, though MIR light cannot interact with AuNP electrons as the interaction with conventional SPR.

Remarkably, this study is performed on a scale relative to the wavelength of light. Thereby, such unique subwavelength manipulation together with localization capability is expected to extend the applications beyond the MIR detection and holds promising potential for advancements in biosensing and nanophotonics in UV/visible ranges.

## Supporting Information Available

Simulation and analysis methods for AuNPs and nanocavities; FDTD simulation parameters (vacuum environment, refractive index, dipole arrangement); coupling effect of dipoles and light field intensity distribution (Figure S1); calculation formulas for resonance conditions, phase shift compensation, and interference phenomena; and nanoscale resonance effects of AuNPs on mid-infrared light confinement (PDF)
