# Supplementary material for: Cavity Effect of Gold Nanoparticles on Mid-Infrared Light
Source: ACS Omega. 2025 Mar 21;10(12):12163–9. doi: 10.1021/acsomega.4c10454 (PMC11966571; doi:10.1021/acsomega.4c10454)
Supplement: Supplementary file 1 — ao4c10454_si_001.pdf [file ao4c10454_si_001.pdf]

Cavity effect of gold nanoparticles on mid-infrared light

Wenjie Yu[1], Cunliang Yang[1], He Min[1], Haipeng Liu[1], Yufeng Ma[1], Zhiheng Yu[1,2], Shuo Yuan[1], Heshuang Dong[1], Ke Wang[1], Bo Song[1*], Jijun Feng[1*]

[1]*Shanghai Key Laboratory of Modern Optical System, Engineering Research Center of Optical Instrument and System (Ministry of Education), School of Optical-Electrical and Computer Engineering, University of Shanghai for Science and Technology, Shanghai 200093, China*

[2]*The Key Laboratory of Medical Electronics and Digital Health of Zhejiang Province, Jiaxing Nanhu University, Jiaxing, Zhejiang 314001, China.*

[*]**Corresponding Authors**: fjijun@usst.edu.cn, bsong@usst.edu.cn

Supplementary Figures

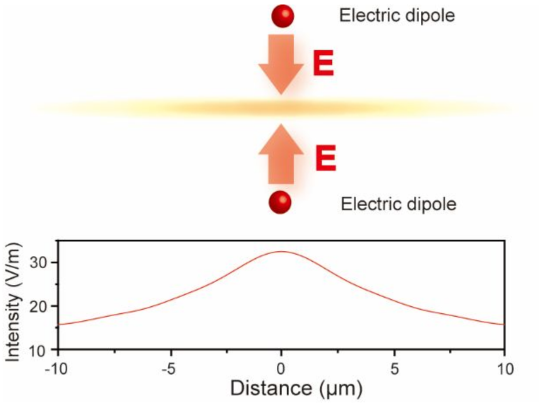

Fig. S1. Light field generated by two point-sources (red balls) for simulating the MIR light released by ATP hydrolysis.

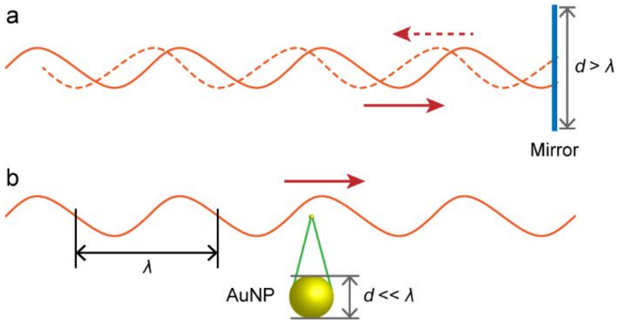

Fig. S2. Effect of an object on the propagation of light wave. a) The light is majorly reflected when the object dimension (*d*) is larger than the wavelength (*λ*), e.g., a mirror. **b)** When the dimension *d* << *λ*, e.g., an AuNP, the light cannot "see" the object, and will bypass it, continuing to propagate along its original trajectory.

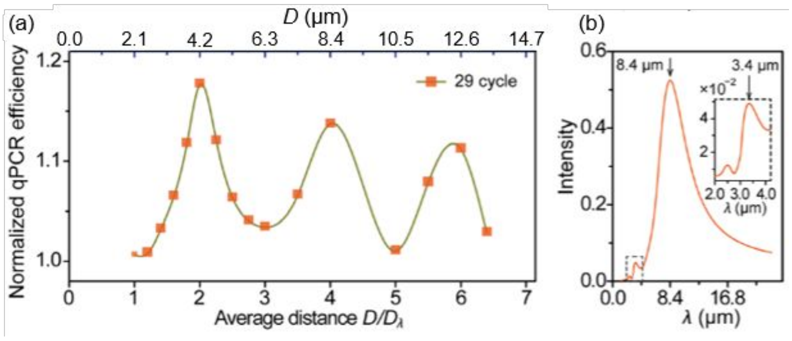

Fig. S3. Periodicity of the qPCR-efficiency oscillation with respect to the average inter-AuNP distance. (a) Periodicity of qPCR-efficiency oscillation with respect to the average distance D after 29th amplification cycle. The values, are normalized to the control (without adding AuNPs). (b) Fourier spectrum with respect to *λ*. Inset: the zoomed view for minor peaks.

The static distribution of AuNP nanoparticles in a colloid

The surface charges of AuNPs enable them to evenly distribute with a dynamically stable structure, as indicated by both theoretical and experimental studies of colloidal metal NPs **[1-4]**. The radial distribution probability $P(r)$ (not the radial distribution function $g(r)$, as proportional to $P(r)/r$) is shown in **Fig. S4**. Two primary peaks, P1 and P2, are always located at the distances $r = d$ and D, respectively, with a ratio of $D/d \approx 1.9$. The nearest distance d of AuNPs can be determined by the AuNP concentration (C) with an equation $d = (N_A C)^{-1/3}$, where N_A means the Avogadro's constant. Although AuNPs randomly diffuse in the colloidal solution due to the Brownian motion, the timescale (microseconds) of the diffusion is much larger than that (picoseconds) of bond breaking/forming in the reaction. Hence, the distribution of AuNPs in the solution can be considered relatively static for the PCR reaction, and thus the AuNP-cavity can be employed for our study.

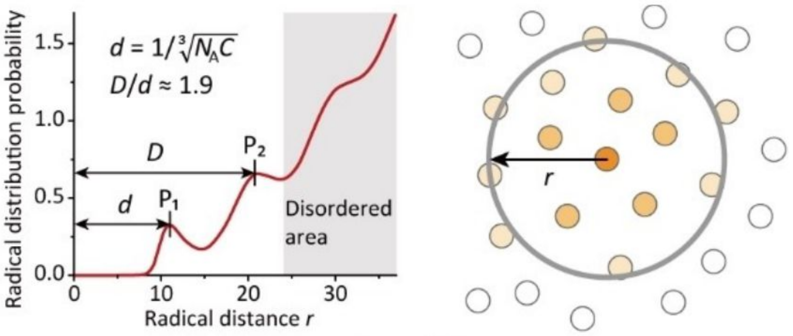

Fig. S4. Radical distribution probability of colloidal metal nanoparticles.

FDTD simulation and analysis methods

In our simulations, we used the frequency domain finite difference method which is directly derived from Maxwell's equations. By spatially discretizing the frequency domain form of Maxwell's equations, we formulated a system of algebraic equations and solved them to obtain the electromagnetic field distribution at specific frequencies. Specifically, the frequency domain form of Maxwell's equations includes:

$$\nabla \times E = j\omega\mu H \quad \textbf{(Eq. S1)}$$

$$\nabla \times H = -j\omega\epsilon E \quad \textbf{(Eq. S2)}$$

Here, E and H represent the electric and magnetic fields, respectively, ω is the angular frequency, and μ and ϵ are the permeability and permittivity of the medium, respectively. By applying the finite difference method to discretize these partial differential equations in space, a matrix equation is formed:

$$AE = b \qquad \textbf{(Eq. S3)}$$

In this context, the matrix A contains information about the difference operators and material parameters, the vector E represents the electric field components to be solved, and the vector b includes source terms and boundary conditions. By solving this system of linear equations, the spatial distribution of the electromagnetic field at a specific frequency can be obtained.

Further, the simulations were carried out by the software of Lumerical FDTD solutions v.8.15.736. The nanocavity structure based on AuNPs (AuNP-cavity) is shown in (**Fig. S1**. To simplify our simulations, we applied a vacuum as the surrounding environment, setting the medium's refractive index (n_g) to 1. Two electric dipoles were positioned symmetrically at a height of ±1 μm, simulating the light source model generated by ATP hydrolysis. The light thus propagated along the x-axis, with peak energy located at the center, decreasing in both directions along the x-axis. The emission wavelength of the electric dipoles was labeled λ. The one-dimensional linear frequency-domain field and power monitor was employed to measure this light field intensity in the nanocavity, with the detection range limited to the dipole emission band λ and the distance between two AuNPs was set to L. Using the finite difference time domain (FDTD) method in two-dimensional mode, we calculated the light field intensity distribution within the AuNP-cavity, exploring nanoscale resonance phenomena within the cavity.

The basic condition for resonance in an optical resonator is that light must undergo multiple reflections, with the phase difference between the two beams at the output satisfies $\Delta\emptyset = 2q\pi$ (where q = 1, 2, 3, …) to generate interference within the cavity, leading to stable enhancement of light resonance. Therefore, in the case of a resonator (i.e., cavity) length of L and a surrounding medium refractive index n_g, the cavity length must satisfy the following equation (1) for resonance to occur:

$$\frac{2\pi}{\lambda}2Ln_g = 2q\pi,$$

$$L = \frac{\lambda q}{2n_g}. \quad \textbf{(Eq. S4)}$$

The surrounding medium refractive index n_g was set at 1 in our simulations. Thus, the cavity length condition was $L = \lambda q/2$ (where q = 1, 2, 3…). By adjusting the distance between AuNPs, we achieved the condition of nanocavity length $L = \lambda q/2$ and identify the position of maximum resonance peak. When the cavity length is an integer multiple of $\lambda/2$, an AuNP-based resonance cavity is formed, confining the light energy within the cavity and reducing the radiation dissipation.

In contrast to conventional resonators, the coupling light source in our simulations was positioned at the center. When light transitions from a low-refractive index medium to a high-refractive index medium (e.g., AuNPs), a phase shift of π, equivalent to half-wavelength loss, occurs. Therefore, under certain cavity length conditions, interference arises between the emitted light and the light reflected from the cavity walls. This interference phenomenon may affect the observation of the resonant optical field within the cavity. To compensate for half-wavelength loss and satisfy the phase difference condition $\Delta\emptyset = 2q\pi$ (where q = 1, 2, 3 …), an additional phase difference of π must be included. Therefore, the relationship between the centrally coupled light source and the AuNPs on both sides that generates interference can be described by the following equation:

$$\frac{2\pi}{\lambda} 2L' n_g = 2q\pi + \pi$$

$$L' = \frac{\lambda(2\mathrm{q}+1)}{4n_g} \quad \textbf{(Eq. S5)}$$

Because n_g = 1, we can derive $L' = \frac{\lambda(2\mathrm{q}+1)}{4}$ where q = 1, 2, 3 …. When the distance from the light source to the cavity wall is $L' = \frac{\lambda(2\mathrm{q}+1)}{4}$, the position of the maximum interference peak can be determined by adjusting the distance between the coupling light source and AuNPs.

Combining **(Eq. S4)** and **(Eq. S5)**, we can conclude that when the cavity length satisfies an integer multiple of the light source wavelength, the interference between

the light source and the AuNP on one side does not affect the resonance caused by the light source reflections within the nanocavity.

Reference

1. Yang, Y.; Peng, D.; Gu, Z.; Jiang, L.; Song, B. AuNP‐Modulated qPCR: An Optimized System for Detecting MIR Biophotons Released in DNA Replication. *Chem. Eur. J.* **2023**, *29*, e202203513.

2. Chopada, R.; Sarwate, R.; Kumar, V. Effect of Mild to Extreme pH, Temperature, and Ionic Strength on the Colloidal Stability of Differentially Capped Gold Nanoparticles. *J. Mol. Struct.* **2025**, *1323*, 140751.

3. Yeomans-Reyna, L.; Acuña-Campa, H.; De Jesús Guevara-Rodríguez, F.; Medina-Noyola, M. Self-Consistent Theory of Collective Brownian Dynamics: Theory versus Simulation. *Phys. Rev. E* **2003**, *67*, 021108.

4. Ojeda-Mendoza, G. J.; Moncho-Jordá, A.; González-Mozuelos, P.; Haro-Pérez, C.; Rojas-Ochoa, L. F. Evidence of Electrostatic-Enhanced Depletion Attraction in the Structural Properties and Phase Behavior of Binary Charged Colloidal Suspensions. *Soft Matter* **2018**, *14*, 1355–1364.
